# Canagliflozin synergises with serine restriction mediating anti-leukaemic effects in T-cell acute lymphoblastic leukaemia

**DOI:** 10.1016/j.molmet.2025.102275

**Published:** 2025-10-19

**Authors:** Fernando M. Ponce-Garcia, Yasmin R. Jenkins, Victoria D. Assmann, Silpita Paul, Nitesh D. Sharma, Catherine Moore, Eric H. Ma, Paraskevi Diamanti, Marc Hennequart, Julianna Blagih, Le Le, Benjamin J. Jenkins, Sophie Rouvray, James G. Cronin, Russell G. Jones, Marc Mansour, Allison Blair, Christina Halsey, Ksenia Matlawska-Wasowska, Daniel Herranz, Emma E. Vincent, Nicholas Jones

**Affiliations:** 1 Institute of Life Science, Swansea University Medical School, Swansea University, SA2 8PP, United Kingdom; 2 Wolfson Wohl Cancer Research Centre, School of Cancer Sciences, College of Medical Veterinary and Life Sciences, University of Glasgow, Glasgow, United Kingdom; 3 Department of Cell, Developmental and Integrative Biology, University of Alabama at Birmingham, Birmingham, AL, USA; 4 Department of Metabolism and Nutritional Programming, Van Andel Institute, Grand Rapids, MI, USA; 5 Cellular and Molecular Medicine, University of Bristol, Biomedical Sciences Building, Bristol, BS8 1TD, United Kingdom; 6 NHS Blood and Transplant, Filton, Bristol, BS34 7QH, United Kingdom; 7 The Francis Crick Institute, 1 Midland Road, London, NW1 1AT, United Kingdom; 8 Namur Research Institute for Life Sciences (NARILIS), Molecular Physiology Unit (URPHYM), University of Namur, Namur, Belgium; 9 University of Montreal, Maisonneuve-Rosemont Hospital Research Centre, 5414 Assomption Blvd, Montreal, H1T 2M4, Canada; 10 Rutgers Cancer Institute of New Jersey, Rutgers University, New Brunswick, NJ, USA; 11 Department of Haematology, Cancer Institute, University College London, London, United Kingdom; 12 Department of Pharmacology, Robert Wood Johnson Medical School, Rutgers University, Piscataway, NJ, USA; 13 Department of Pediatrics, Robert Wood Johnson Medical School, Rutgers University, Piscataway, NJ, USA; 14 School of Translational Health Sciences, Dorothy Hodgkin Building, University of Bristol, Bristol, BS1 3NY, United Kingdom; 15 Integrative Epidemiology Unit, School of Population Health Science, University of Bristol, Bristol, BS8 2BN, United Kingdom

**Keywords:** Leukaemia, Serine, Glycine, Metabolism, T-ALL, Canagliflozin

## Abstract

T-cell acute lymphoblastic leukaemia (T-ALL) is a haematological malignancy commonly driven by *NOTCH1* activating mutations. A concomitant feature associated with *NOTCH1* mutations is heightened oxidative metabolism enabling the exponential proliferation of T-ALL blasts. As such, targeting mitochondrial metabolism in T-ALL is an attractive therapeutic avenue. Related to this, canagliflozin (cana), is an FDA-approved sodium glucose co-transporter 2 inhibitor with known off-target effects on complex I and glutamate dehydrogenase, but its potential anti-leukaemic effects remain unexplored. Here, we show that cana possesses potent anti-leukaemic effects underpinned by proliferative defects, cell cycle disruption and apoptosis. These anti-leukaemic effects driven by cana, are attributed to a perturbed tricarboxylic acid (TCA) cycle and mitochondrial metabolism, and elevated mitochondrial ROS. Proteomic analysis revealed that cana treatment resulted in a compensatory increase in the expression of ATF4 targets, including upregulation of serine biosynthesis pathway and one-carbon metabolism enzymes. As such, restriction of serine and glycine synergized with cana treatment, further enhancing its anti-leukaemic effects. Collectively, our study reveals a cana-driven metabolic vulnerability that can be further exploited via dietary manipulation to treat T-ALL.

## Highlights

• Canagliflozin impairs human T-ALL proliferation, and impacts viability, extending to a broad range of genetic subtypes.
• Cana reprogrammes the proteome in T-ALL leading to compensatory increases in serine, glycine and one carbon metabolism.
• Restriction of serine/glycine synergistically complemented canagliflozin treatment by heightening T-ALL apoptosis.

## 1 Introduction

T-ALL is a haematological cancer affecting the development of immature T-cells. Whilst initially treatable, chemoresistance frequently occurs (between 20 and 50 %), increasing disease aggressiveness [1,2]. Many cancers are characterised by upregulated aerobic glycolysis; however, T-ALL blasts balance upregulation of both glycolysis and oxidative phosphorylation (OXPHOS) to support their metabolic program – indicating a key role for mitochondria [2,3]. T-ALL blasts are heavily dependent on their mitochondria for the generation of biosynthetic intermediates and energy-rich molecules in the form of adenosine triphosphate (ATP) [2], and hence, mitochondria are an attractive therapeutic target [2]. Indeed, pharmacological inhibition of mitochondrial complex I with rotenone or IACS-010759 elicit promising anti-leukaemic effects in T-ALL [4,5]. Furthermore, a recently explored mitochondrial uncoupling drug, MB1-47, caused cytotoxic effects, ultimately reducing proliferation in T-ALL blasts [6], however its safety profile has not yet been tested in humans.

Whilst targeting mitochondrial metabolism remains an attractive therapeutic avenue across many cancers, difficulties have arisen due to toxicity resulting in severe, intolerable side effects [7]. Recently, a phase I clinical trial investigating IACS-010759 in advanced solid tumours and acute myeloid leukaemia was halted due to unacceptable drug-associated toxicities [8]. Therefore, the repurposing of well-tolerated existing drugs that have well-established safety profiles and are already used to treat metabolic disorders, such as diabetes, is an attractive alternative to exploit metabolic vulnerabilities for therapeutic gains.

Sodium glucose co-transporter 2 (SGLT2) inhibitors, or gliflozins, are a relatively new class of drugs used to treat people with type 2 diabetes (T2D) [9]. Gliflozins such as canagliflozin (cana) and dapagliflozin (dapa) inhibit sodium glucose co-transporter 2 (SGLT2 or *SLC5A2*) to prevent glucose reabsorption in the kidneys and improve glycaemic control [10,11]. The on-target effects of gliflozins (SGLT2 inhibition), resulting in reduced glucose transport, have already led to investigation of their effects on cancers such as lung adenocarcinoma, colon adenocarcinoma, hepatocellular carcinoma and adult T-cell leukaemia, where tumour growth was impeded [[12], [13], [14], [15]]. Additionally, the cardioprotective potential of gliflozins in the setting of anti-cancer drug-induced cardiotoxicity has also been highlighted [16,17]. Specifically, dapa and empagliflozin (empa) have been shown to protect against the cardiotoxic effects of the tyrosine kinase inhibitor ponatinib, by reducing endothelial cell senescence, with empa also restoring autophagy in cardiomyocytes [18,19]. Aside from SGLT2 inhibition, cana, as opposed to other gliflozins, possesses interesting off-target inhibitory effects on complex I and glutamate dehydrogenase (GDH) [20] in the mitochondria.

A number of studies have demonstrated the anti-cancer properties of cana in solid tumour settings [21,22]. However the potential anti-leukaemic effects of cana in T-ALL have not been considered to date. Since gliflozins are already prescribed safely to people with T2D and have biologically relevant effects on metabolism, they could be exploited as a monotherapy or in combination to treat oxidative malignancies such as T-ALL.

Here we report that cana possesses broad anti-leukaemic effects in T-ALL, irrespective of NOTCH1 status. These anti-leukaemic effects stem from a suppressed oxidative metabolism attributed to the off-target effects of cana on complex I and GDH. Specifically, we show that cana rewires the global proteome in T-ALL, leading to a compensatory increase in serine, glycine and one-carbon metabolism downstream of ATF4. To further exploit this, we found that serine/glycine restriction enhances the anti-leukaemic effects of cana *in vitro* and *in vivo*. Taken together, we reveal that cana possesses strong anti-leukaemic effects and highlight its potential for therapeutic use in T-ALL.

## 2 Results

### 2.1 Canagliflozin exhibits anti-proliferative and cytotoxic effects in T-ALL

**Figure 1:**
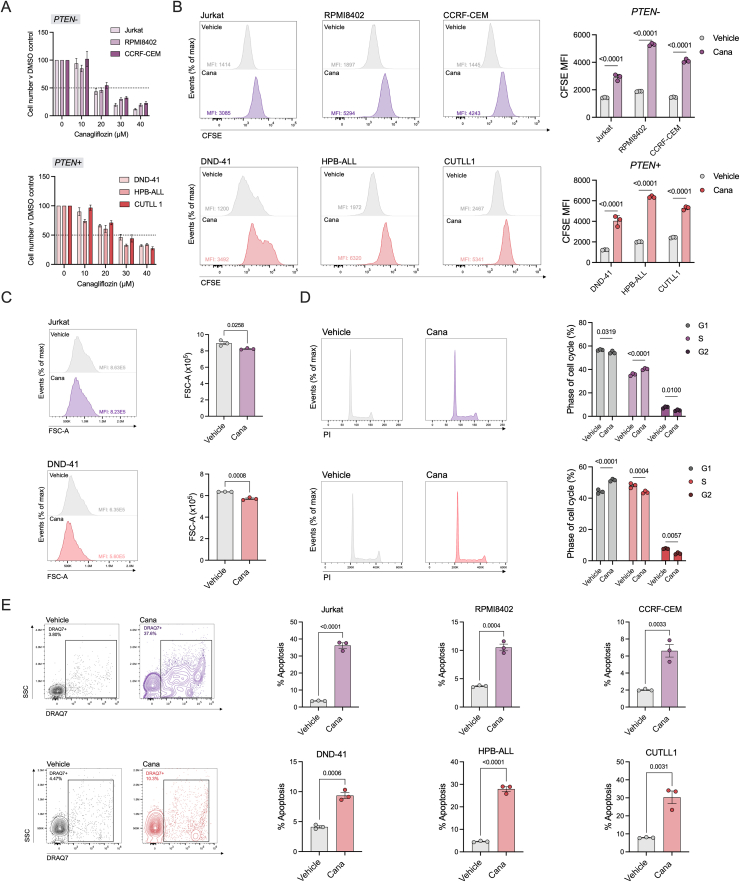
**Canagliflozin reduces cellular proliferation in T-ALL.** Cell proliferation normalised to DMSO vehicle control of PTEN^−^ or PTEN^+^ T-ALL cell lines following treatment with cana at the indicated concentrations for 72 h as (A) cell counts or (B) via CFSE staining. (C) Cell size (FSC-A) measured using flow cytometry after treatment with cana (30 μM) for 72 h. (D) Cell cycle distribution analysis of Jurkat and DND-41 cells following treatment with DMSO vehicle control or 30 μM cana for 72 h, determined by flow cytometry using propidium iodide DNA staining. (E) Percentage cell death following treatment with DMSO vehicle control or 30 μM cana for 72 h, measured by flow cytometry using DRAQ7. Representative flow cytometry contour plots of Jurkat (purple) and DND-41 (red) cells showing DRAQ7+ gating strategy. Data are representative of n = 3 and expressed as mean ± SEM. Statistical analysis was carried out using an unpaired T test (C, E) or a two-way ANOVA with Sidak's (B, D) multiple comparisons test.

**Supplementary Figure 1:**
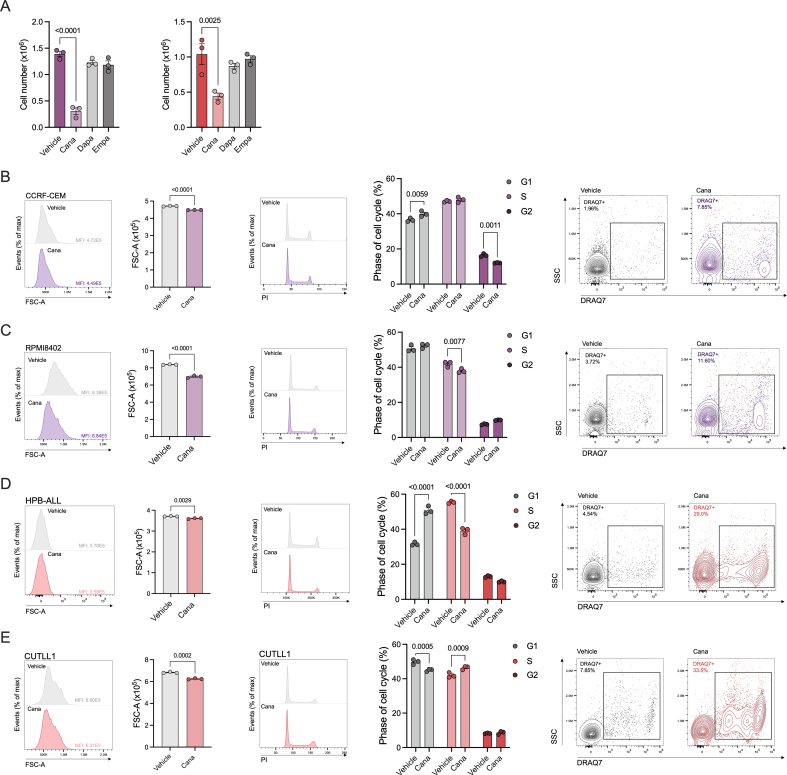
**Canagliflozin promotes anti-leukaemic effects in T-ALL.** (A) Cell proliferation of Jurkat and DND-41 T-ALL cells following treatment with SGLT2 inhibitors cana, dapa or empa (30 μM). Cell size (FSC-A), cell cycle or cell viability measured using flow cytometry after 72 h of cana (30 μM) in (A) CCRF-CEM, (B) RPMI8402, (C) HPB-ALL or (D) CUTLL1 T-ALL cells. Data are representative of n = 3 and expressed as mean ± SEM. Statistical analysis was carried out using a one way ANOVA (SGLT2 inhibitors), an unpaired T test (cell size) or a two-way ANOVA with Sidak's multiple comparisons test (cell cycle).

**Supplementary Figure 2:**
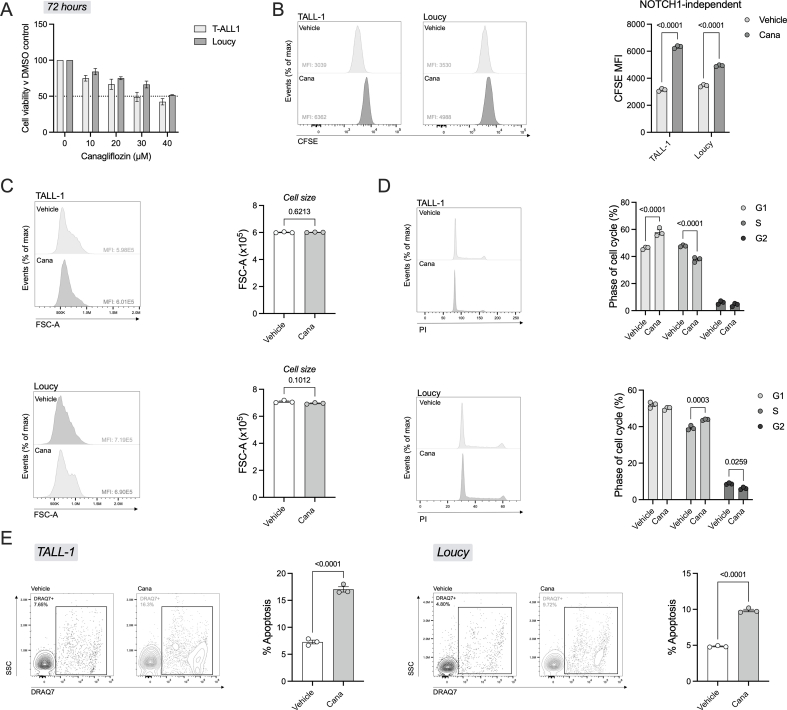
**Canagliflozin promotes anti-leukaemic effects in NOTCH1-indepedent T-ALL.** Cell proliferation normalised to DMSO vehicle control of NOTCH1-independent T-ALL cell lines, T-ALL1 or Loucy, following treatment with cana at the indicated concentrations or (B) 30 μM for 72 h as (A) cell counts or (B) via CFSE staining. (C) Cell size (FSC-A) measured using flow cytometry after 72 h of cana (30 μM). (D) Cell cycle distribution analysis of TALL-1 or Loucy cells following treatment with DMSO vehicle control or 30 μM cana for 72 h, determined by flow cytometry using propidium iodide DNA staining. (E) Percentage of cell death following treatment with DMSO vehicle control or 30 μM cana for 72 h, measured by flow cytometry using DRAQ7. Representative flow cytometry contour plots of TALL-1 and Loucy cells showing DRAQ7+ gating strategy. Data are representative of n = 3 and expressed as mean ± SEM. Statistical analysis was carried out using an unpaired T test (C, E) or a two-way ANOVA with Sidak's multiple comparisons test (B, D).

**Supplementary Figure 3:**
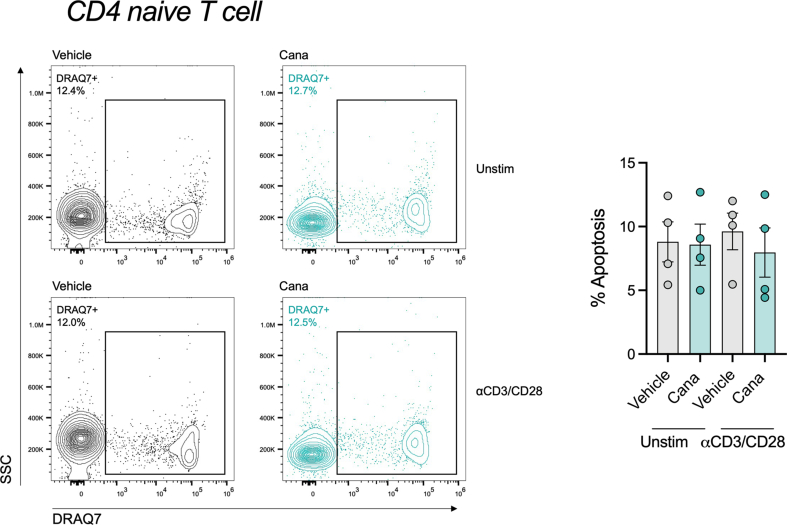
**Canagliflozin does not impact the viability of human CD4^+^ naïve T-cells.** CD4^+^ naïve T-cells isolated and activated with anti-CD3 (2 μg/mL) and anti-CD28 (20 μg/mL) for 24 h in the presence or absence of cana (30 μM). Viability assessed using DRAQ7. Representative flow cytometry contour plots of CD4^+^ naïve T-cells showing DRAQ7+ gating strategy. Data are representative of n = 4 and expressed as mean ± SEM.

We sought to better understand the effects of canagliflozin (cana) on multiple NOTCH1-mutated human T-ALL cell lines harbouring additional mutational backgrounds (broadly classified as PTEN^−^, *Jurkat, RPMI8402* and *CCRF-CEM* or PTEN^+^, *DND-41, HPB-ALL and CUTLL1*). We employed a dose range of cana between 0 and 40 μM, in line with previous studies [20,22]. Here, independent of mutational background, cana suppressed T-ALL cell proliferation in a dose-dependent manner (Figure 1A and B). These anti-proliferative effects of cana were not extended to two other SLGT2 inhibitors; dapagliflozin (dapa) and empagliflozin (empa) (Supplementary Figure 1A). To further examine the anti-leukaemic effects of cana, we selected one representative PTEN^−^ (Jurkat) and PTEN^+^ (DND-41) cell line. Consistent with decreased proliferation, cana also significantly reduced cell size, perturbed cell cycle and increased cell death indicative of anti-leukaemic effects (Figure 1C and E). Interestingly, these observations were largely consistent on the expanded T-ALL cell line panel (Supplementary Figure 1B and E), and on two NOTCH1-independent leukaemic cell lines (*TALL-1 and Loucy*; Supplementary Figure 2A–E). Importantly, we observed no decrease in cell viability when treating primary human CD4+ naïve T-cells with equimolar concentrations of cana (Supplementary Figure 3). Taken together, these data demonstrate that cana exerts anti-leukaemic effects on T-ALL irrespective of mutational background.

### 2.2 Canagliflozin impairs oxidative metabolism in T-ALL

**Figure 2:**
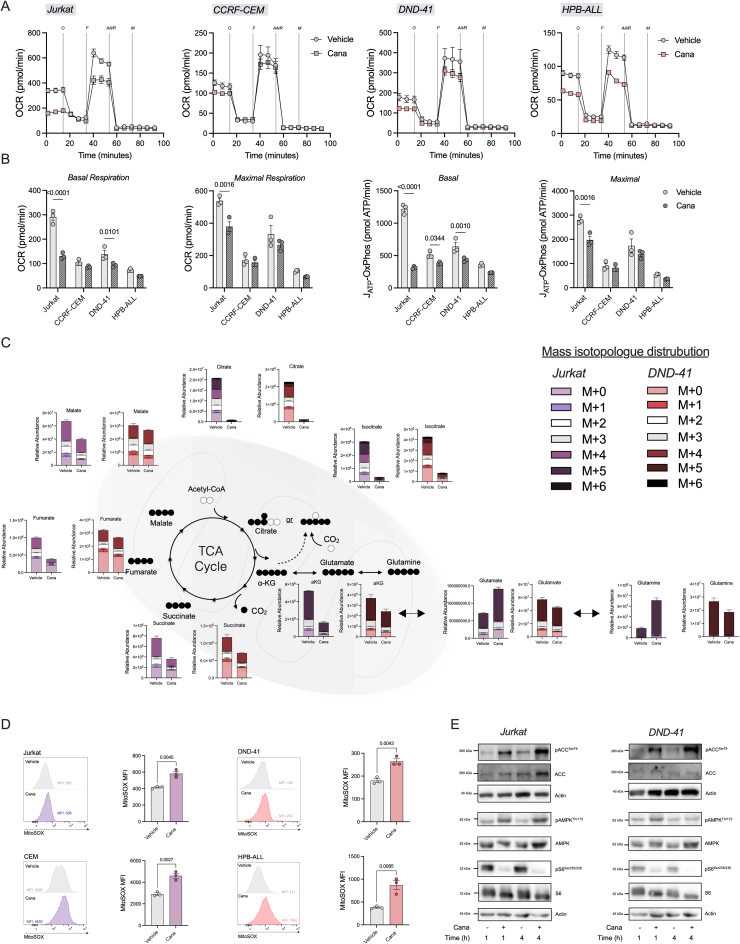
**Canagliflozin suppresses oxidative metabolism in T-ALL.** (A) Jurkat, CCRF-CEM, DND-41 or HPB-ALL cells treated with DMSO vehicle control or cana (30 μM) for 72 h. Seahorse extracellular flux analysis measured via oxygen consumption rate (OCR) using injections of oligomycin, FCCP, antimycin A/rotenone (all 1 μM) and monensin (20 μM). (B) Oxidative parameters; basal respiration, maximal respiration, basal or maximal ATP production (J_ATP_) from OXPHOS assessed. (C) Stable isotope tracing of uniformly labelled ^13^C_5_-glutamine into the TCA cycle. Relative abundance of individual mass isotopologues derived from ^13^C_5_-glutamine towards TCA cycle metabolite pools. (D) Mitochondrial ROS assessed using MitoSOX in T-ALL cells treated as (A), including representative flow cytometry plots. (E) Immunoblot of pACC^Ser79^, pAMPK^Thr172^, AMPK, pS6^Ser235/236^ and S6, with actin used as loading control, in Jurkat and DND-41 cells treated for 1 and 4 h with or without cana (30 μM). Data are representative of n = 3 independent experiments and expressed as mean ± SEM. Statistical analysis was carried out using a two-way ANOVA with Sidak's (B) multiple comparisons test or an unpaired T test (D).

**Supplementary Figure 4:**
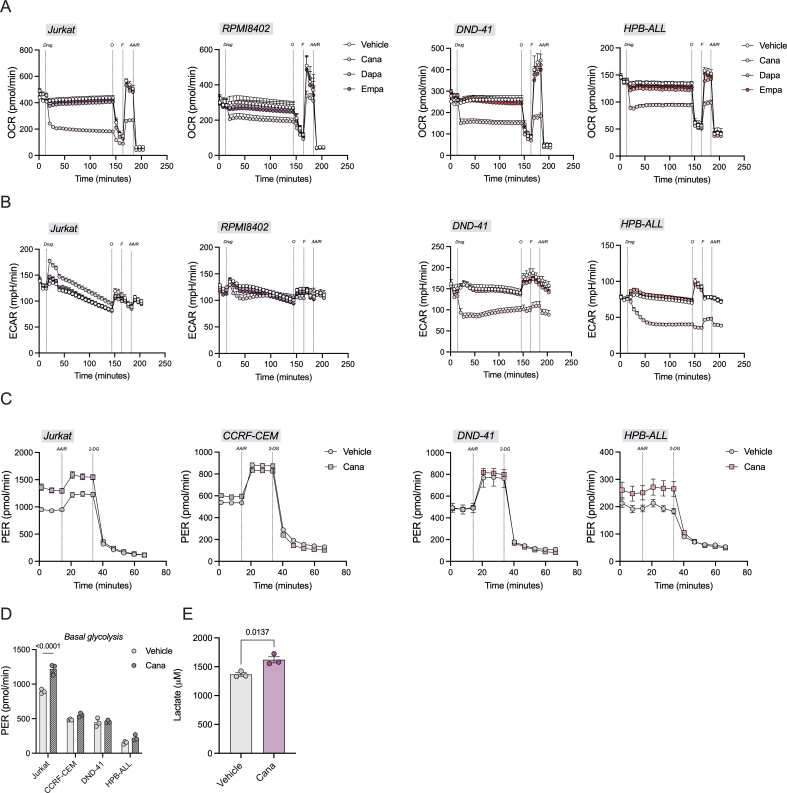
**Canagliflozin impacts the metabolic profile in T-ALL.** Seahorse extracellular flux analysis investigating the (A) oxygen consumption rate (OCR; oxidative phosphorylation) or (B) extracellular acidification rate (ECAR; glycolysis) of Jurkat, RPMI8402 (PTEN-) and DND-41, HPB-ALL (PTEN+) cell lines exposed to gliflozins. Gliflozins (canagliflozin, dapagliflozin or empagliflozin) were administered at 30 μM for 2 h, after which a mitochondrial stress test was performed using oligomycin (1 μM), FCCP (1 μM) and antimycin A/rotenone (1 μM). (C) Glycolytic rate assay calculated using proton efflux rate (PER) of Jurkat, CCRF-CEM, DND-41 or HPB-ALL after 72 h of culture in the presence or absence of cana (30 μM) with (D) Basal glycolysis assessed. (E) Extracellular lactate levels of Jurkat cell supernatant after 24 h of culture with cana (30 μM). Data are representative of n = 3–4 independent biological experiments and expressed as mean + or ± SEM. Statistical analysis was carried out using a two-way ANOVA with Bonferroni's (D) multiple comparisons test or an unpaired t-test (E).

Given the established inhibitory effects of cana on GDH and complex I [23], we next investigated whether cana treatment disrupted cellular metabolism in T-ALL. To this end, we cultured a range of T-ALL cell lines in the presence of cana and assessed the impact on cellular metabolism using extracellular flux analysis. Cana treatment immediately decreased the oxygen consumption rate (OCR) in all cell lines analysed, with a variable response in the extracellular acidification rate (ECAR; Supplementary Figure 4A and B). Interestingly, no metabolic perturbation was exhibited using alternative SGLT2 inhibitors, dapa or empa, that do not possess off-target effects. Next, we assessed the longer-term exposure of cana on the metabolic phenotype of T-ALL cells. Broadly, 72 h cana treatment suppressed oxidative metabolism resulting in a compensatory increase (Jurkat) or maintenance (CCRF-CEM, DND-41 and HPB-ALL) in basal glycolysis levels (Figure 2A and B; Supplementary Figure 4C and D). This increase in basal glycolysis levels in Jurkat cells is in agreement with increased extracellular lactate as measured in the supernatant (Supplementary Figure 4E). Notably, the T-ALL cells where cana had the largest impact on oxidative metabolism, were the cells that had a higher level of cell death (Figure 1E).

**Supplementary Figure 5:**
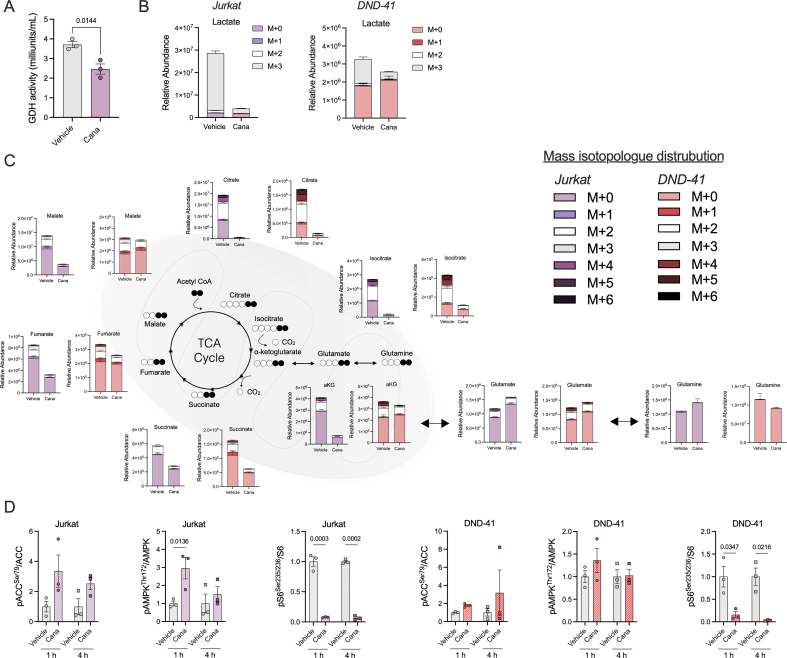
**Canagliflozin impairs GDH activity and glucose metabolism.** (A) Glutamate dehydrogenase (GDH) activity in Jurkat cells treated with cana (30 μM) for 24 h. (B) Stable isotope tracing analysis of Jurkat and DND-41 cells treated with or without cana (30 μM) for 24 h in the presence of ^13^C_6_-glucose for the final 8 h. Relative abundance of the mass isotopologues of lactate in Jurkat (purple) or DND-41 (red). (C) Jurkat and DND-41 treated as (B) with relative abundance of TCA cycle associated metabolites. (D) Densitometry analysis of pACC^Ser79^/ACC, pAMPK^Thr172^/AMPK and pS6^Ser235/236^/S6 normalised to actin and relative to control. Data are representative of n = 3 independent experiments. Data are expressed as mean + or ± SEM. Statistical analysis was carried out using a two-way ANOVA with Bonferroni's (D) multiple comparisons test.

To gain further insight into the precise effect of cana on cellular metabolism, we performed stable isotope tracing analysis (SITA) with a uniformly-labelled ^13^C_5_-glutamine (Gln). Jurkat and DND-41 cells cultured in the presence of cana for 24 h, followed by an 8-hour pulse of ^13^C_5_-Gln, revealed a significant reduction of glutamine-derived alpha-ketoglutarate and downstream metabolites (Figure 2C). These data align cana's inhibitory effect on GDH, which is further supported by reduced enzymatic activity of GDH in Jurkat cells (Supplementary Figure 5A). Moreover, this marked reduction in glutamine incorporation highlights the importance of this two-pronged approach – combining GDH inhibition with complex I inhibition – by which cana offers enhanced modulation of mitochondrial metabolism compared to other complex I inhibitors.

Since mitochondrial metabolism is equally upregulated in T-ALL cells [4], we next evaluated the effect of glucose utilization in the presence of cana. SITA with ^13^C_6_-glucose in cana-treated and untreated T-ALL cells (Jurkat and DND-41) revealed lower intracellular lactate levels (Supplementary Figure 5B) followed by suppressed ^13^C incorporation into the TCA cycle, in line with the glutamine tracing data (Supplementary Figure 5C).

As cana has been reported to inhibit complex I [23], we assessed levels of mitochondrial ROS (mROS) using the mitochondrial fluorescent probe, mitoSOX. Here, upon cana treatment, T-ALL cell lines exhibit significantly increased mROS levels (Figure 2D), consistent with alternative complex I inhibitors promoting heightened mROS [24]. Given the profound effect that cana elicits on cellular metabolism, we next investigated two critical metabolic nodes, AMPK and mTOR. Here, upon cana treatment activation of downstream AMPK target, ACC, was increased, with a concomitant reduction in the phosphorylation of downstream mTOR target, S6 (Figure 2E and Supplementary Figure 5D). Collectively, these data demonstrate that cana suppresses oxidative metabolism leading to downstream consequences on cellular signalling.

### 2.3 Canagliflozin modulates the T-ALL proteome

**Figure 3:**
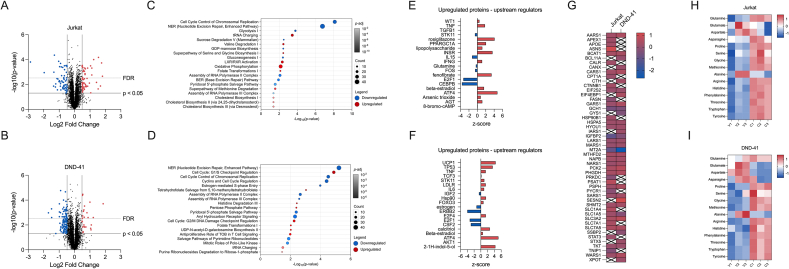
**Canagliflozin remodels the T-ALL proteome.** Differential protein expression of label-free mass spectrometry in cana-treated (A) Jurkat or (B) DND-41 cells versus vehicle control (DMSO). Blue and red data points represent downregulated and upregulated genes, respectively. Proteins with an adjusted p-value <0.05 and log_2_ fold-change >0.585 were considered differentially expressed. (C–D) Ingenuity pathway analysis (IPA) of canonical pathways based on significantly downregulated and upregulated proteins in (C) Jurkat and (D) DND-41 cells. Top 10 enriched pathways are shown. (E–F) Analysis of predicted upstream regulators in IPA based on significantly upregulated proteins in (E) Jurkat and (F) DND-41 cells. (G) Proteins associated with the predicted ATF4 signature identified from upstream regulator analysis. (H–I) Intracellular amino acid content of Jurkat and DND-41 cells treated with cana (30 μM) for 24 h. Data are representative of n = 3 independent experiments.

Thus far we have demonstrated that cana possesses anti-leukaemic properties on a range of T-ALL lines, with significant impact on the AMPK/mTOR axis. Therefore, we next took an approach to elucidate the global effects of cana treatment on the T-ALL proteome using tandem mass tagging quantitative proteomics. T-ALL cells (Jurkat and DND-41) were treated with cana for 24 h, where we observed minimal cell death, thus carrying out our proteomic analysis on the live cell proteome. Here, we were able to detect a total of 8955 proteins in both the Jurkat and DND-41 cell lines (Figure 3A and B). Cana treatment resulted in 142 upregulated proteins and 68 downregulated proteins in Jurkat cells (Figure 3A) and 38 upregulated proteins and 158 downregulated in the DND-41 cells (Figure 3B). To further probe the biological impact of these changes, we employed ingenuity pathway analysis (IPA). Here, IPA revealed that, unsurprisingly, the majority of downregulated canonical pathways were focused around cellular proliferation defects such as *cell cycle control of chromosomal replication, nucleotide excision repair* and *assembly of RNA polymerase II complex* (Figure 3C and D). Conversely, the upregulated canonical pathways involved various aspects of serine, glycine and one carbon metabolism, including *serine and glycine biosynthesis, folate transformations* and *tetrahydrofolate salvage* (Figure 3C and D). Next, we validated predicted upstream regulators in accordance with our proteomic dataset. Here, ATF4 was the most highly predicted regulator for both Jurkat and DND-41 cells (Figure 3E and F). Underscoring the predicted ATF4 signature were proteins associated with amino acid transport (SLC1A4, SLC1A5, SLC3A2, SLC7A1 and SLC7A5), serine and glycine metabolism (PHGDH, PSAT1, PSPH) and one carbon metabolism (MTHFD2, SHMT2; Figure 3G). Given the robust increase in amino acid transporters, we next assessed levels of intracellular amino acids by mass spectrometry. T-ALL cells treated with cana had a remarkable increase in amino acid content in comparison to the vehicle control (Figure 3H and I). Taken together, these data highlight that cana treatment elicits a potential compensatory mechanism geared towards increased amino acid transport and heightened one carbon metabolism.

### 2.4 Canagliflozin increases compensatory serine metabolism

**Figure 4:**
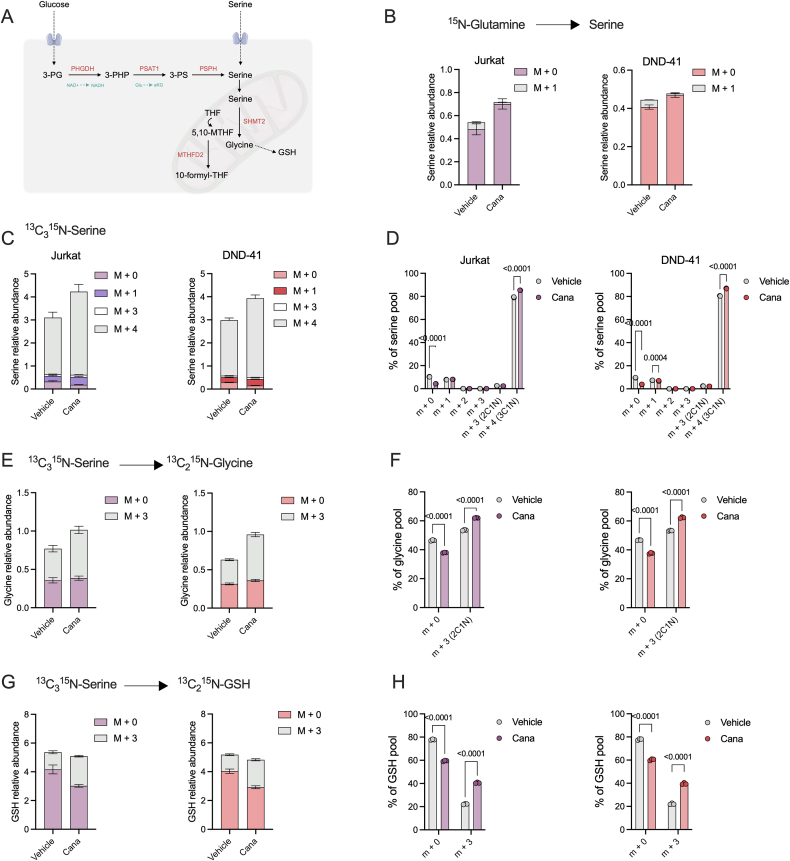
**Canagliflozin increases serine uptake but not *de novo* synthesis.** (A) Schematic of the serine synthesis pathway. (B) Relative abundance of ^15^N_1_-glutamine contribution towards the serine pool in Jurkat and DND-41 cells treated with cana (30 μM) for 24 h. (C) Relative abundance of ^13^C_3_^15^N_1_-serine towards the intracellular pool in Jurkat and DND-41 cells treated as (B). (D) Mass isotopologue distribution (MID) represented as a % of the total intracellular serine pool. Relative abundance and MID of ^13^C_3_^15^N_1_-serine towards the (E–F) glycine or (G–H) glutathione pools in Jurkat and DND-41 cells. Data are representative of n = 3 independent experiments and expressed as mean + or ± SEM.

Our proteomics dataset highlighted a potential compensatory increase in serine, glycine and one carbon metabolism. To initially explore this, we assessed whether the dependence on serine was derived from *de novo* synthesis or uptake. We reasoned that the conversion of glutamate to alpha-ketoglutarate, catalysed by PSAT1, could bypass the inhibition on mitochondrial GDH (Figure 4A). To our surprise, we did not see an increase in m+1 labelling of the serine pool derived from ^15^N-glutamine tracing (Figure 4B). In contrast, when we explored serine uptake using a ^13^C_3_^15^N-serine probe, we observed an increase in intracellular, fully labelled (m+4) serine (Figure 4C and D). This increase in serine uptake resulted in the elevated incorporation of ^13^C_2_^15^N into the downstream glycine pool in Jurkat and DND-41 cells (m+3; Figure 4E and F).

One such use of increased serine/glycine metabolism is conversion towards the antioxidant glutathione (GSH; Figure 4A) as a potential mechanism to counteract the elevated mROS levels. Here, increased ^13^C_2_^15^N labelling in the GSH pool was observed in both Jurkat and DND-41 cells (Figure 4G and H). These data highlight that cana treatment promotes a compensatory increase in serine and glycine metabolism in T-ALL.

### 2.5 Inhibition of serine metabolism enhances the anti-leukaemic effects of canagliflozin

Given the profound increase in serine (S), glycine (G) and one carbon metabolism-associated enzymes accompanied by elevated serine uptake upon cana treatment, we wanted to determine whether targeting serine metabolism could enhance the efficacy of cana. To this end, we employed two approaches, the first using a specific pharmacological phosphoglycerate dehydrogenase (PHGDH) inhibitor, BI-4916, and secondly using media without S/G.

**Figure 5:**
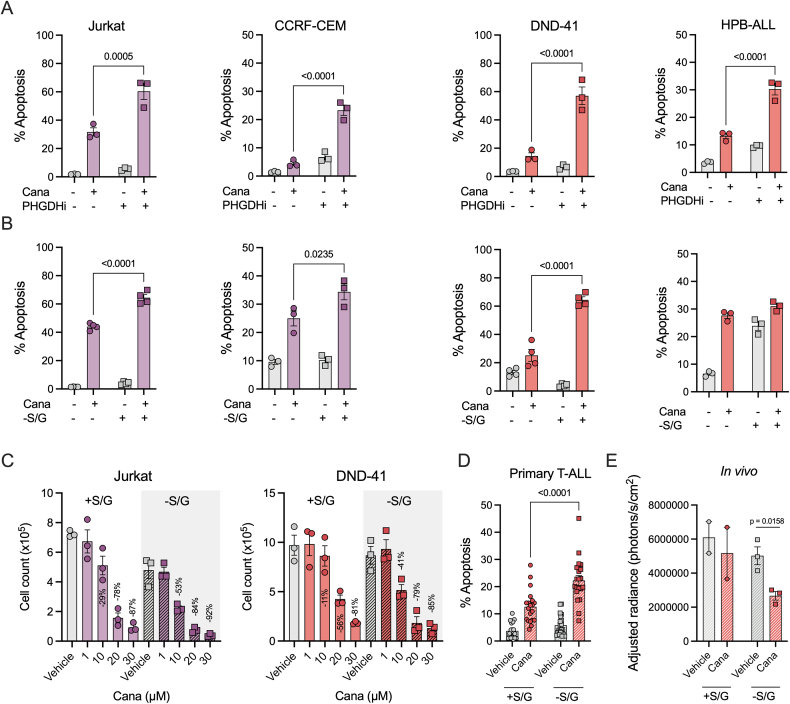
**Canagliflozin synergises with serine and glycine restriction in T-ALL.** (A) Jurkat, CCRF-CEM, DND-41 and HPB-ALL cells treated with BI-4916 for 72 h in the presence or absence of cana (30 μM) with cell viability assessed using DRAQ7. (B) Cells cultured in the presence and absence of cana (30 μM) in complete or serine/glycine depleted media for 72 h (C) Proliferation of Jurkat and DND-41 cells cultured with a titration of cana (0–30 μM) in complete or serine/glycine depleted media. Data are representative of n = 3. (D) Primary T-ALL patient samples (n = 20) cultured with cana (30 μM) in complete or serine/glycine depleted media. (E) IVIS imaging results of day 31. Two mice of the +S/G group had previously reached clinical endpoint, and the corresponding groups were excluded from statistical analysis due to the small samples size. Data are represented as mean ± SEM.

**Supplementary Figure 6:**
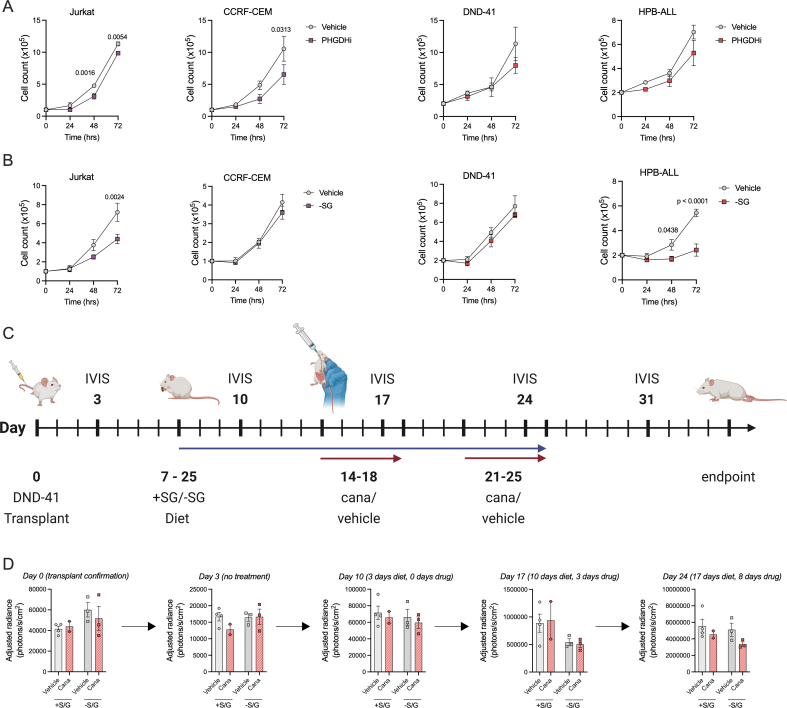
**The effects of PHGDH inhibition or serine/glycine restriction on T-ALL proliferation.** Cell lines Jurkat, CCRF-CEM, DND-41 or HPB-ALL treated for 72 h in the presence or absence of (A) BI-4916 (PHGDH inhibitor; 15 μM) or (B) serine and glycine restriction. Cell proliferation assessed by cell counts. Data are representative of n = 3 independent experiments. Data are expressed as mean ± SEM. (C) Schematic of *in vivo* experimental design. Mice were transplanted on day 0 with DND-41^GFP−Luc^ cells, received the test diet from day 7 to day 25 and were treated with cana or vehicle on days 14–18 and 21 to 25. IVIS imaging was done on days 0, 3, 10, 17, 24 and 31. Mice were culled at clinical endpoint. (D) IVIS imaging results at days 0, 3, 10, 17 and 24. Data are represented as mean ± SEM.

We initially determined the sensitivity of the above approaches in a panel of T-ALL cell lines (Jurkat, CCRF-CEM, DND-41, HPB-ALL). Interestingly, Jurkat and CCRF-CEM cell lines were modestly sensitive to PHGDH inhibition, whereas DND-41 and HPB-ALL were not (Supplementary Figure 6A). In contrast, S/G depletion in Jurkat and HPB-ALL led to impaired proliferation (Supplementary Figure 6B). However, dual treatment with cana and S/G deprivation or PHGDH inhibition drove cell death in all T-ALL lines (Figure 5A and B). Furthermore, S/G restriction even sensitised leukemic cells to lower doses of cana (10–30 μM), resulting in reduced proliferation compared to complete media (Figure 5C). Next, we utilised a small cohort (n = 20) of primary T-ALL samples. Excitingly, treatment with cana increased cell death in comparison to the vehicle control. These anti-leukaemic effects were further enhanced in combination with S/G withdrawal (Figure 5D). Finally, as a proof-of-principle, we aimed to explore whether the dual treatment of cana and S/G free diet in mice could reduce leukaemic burden in a pilot study. For this purpose, 15 male NOD-Scid-γ (NSG) mice were transplanted with DND-41^GFP−Luc +^ cells and randomly assigned to the +S/G vehicle, +S/G cana, -S/G vehicle or -S/G cana treatment groups. After an engraftment period of 7 days, mice were placed on an experimental diet with an adjustment time of 7 days before receiving two blocks of drug treatment for 5 days (Supplementary Figure 6C). Disease progression and leukaemic burden were assessed by IVIS imaging (Supplementary Figure 6D and E). Interestingly, the final imaging at 31 days post-transplant showed a significantly reduced leukaemic burden in mice treated with cana and -S/G diet (Figure 5E). Collectively, these data underscore a promising combinatory therapeutic strategy of S/G restriction and cana treatment.

## 3 Discussion

Targeting the dysregulated metabolic profile in T-ALL is an attractive therapeutic avenue [1,2,5,6]. Indeed, chemotherapies targeting metabolism, such as methotrexate and l-asparaginase, have been the standard-of-care therapy for leukemia [25,26]. Recently, studies have revealed that T-ALL considerably relies on mitochondrial complex I [5,27], whereby therapeutic targeting of complex I highlights a significant metabolic vulnerability [4]. Therefore, investigations into clinically approved drugs that target complex I may offer safer novel therapeutic options in T-ALL treatment. Here, we investigated repurposing the T2D medication, cana, as a therapeutic strategy for T-ALL. Cana was originally developed to inhibit SGLT2, thus improving glycaemic control, and has an established safety profile in humans. Whilst the repurposing of other SGLT2 inhibitors, dapa and empa, has been proposed in the setting of anti-cancer drug-induced cardiotoxicity [[16], [17], [18], [19]], it is cana's off-target effects that make it an attractive therapeutic prospect for T-ALL. Numerous studies have revealed additional off-target effects of cana, specifically on metabolism-associated proteins, complex I and GDH [23,28]. Excitingly, a recent plethora of studies have explored surplus benefits of cana beyond glycaemic control in aging [29], autoimmune [28] and cancer [21,22] settings.

In this manuscript, we demonstrate that cana exerts potent anti-leukaemic effects in T-ALL by reducing proliferation and cell size, perturbing cell cycle and enhancing cellular apoptosis. Importantly, the observed effects were apparent on a panel of human T-ALL cell lines harbouring different mutation signatures and not extended to other SGLT2 inhibitors such as dapa and empa. These anti-leukaemic effects are mediated by the off-target effects of cana on complex I and GDH, resulting in significant suppression of oxidative metabolism concomitant with elevated mROS. Although we observed a compensatory increase or maintenance in basal glycolysis levels – illustrating the metabolic plasticity of T-ALL cells in their ability to balance the upregulation of both glycolysis and OXPHOS – the anti-leukaemic effects of cana were nonetheless bolstered by the dual inhibition of GDH alongside complex I. Here, glutamine incorporation into TCA cycle intermediates was significantly reduced upon cana treatment. Interestingly, there was a more pronounced reduction in citrate compared to other TCA cycle intermediates. This is perhaps indicative of wider metabolic reshaping in these cells, where there is increased ACLY-mediated conversion of citrate to acetyl-CoA, that can fuel lipid associated pathways such as fatty acid synthesis and drive epigenetic remodelling through enhanced histone acetylation [30]. Indeed, this is in line with recent studies that have described a reliance on ACLY activity in T-ALL [31,32].

Strikingly, cana treatment was associated with a compensatory increase in downstream ATF4 targets, including proteins associated with amino acid transport, serine, glycine and one carbon metabolism. Dependence on serine metabolism has previously been reported in T-ALL, with inhibition of PSPH eliciting potent anti-leukaemic effects [33]. In contrast, our observed compensatory increases in ATF4 targets resulted in heightened serine import rather than *de novo* synthesis, fuelling glycine and subsequently, glutathione pools. Unexpectedly, restriction of serine/glycine synergistically complemented cana treatment by heightening T-ALL apoptosis, highlighting the possibility of a dietary intervention alongside cana treatment. Further studies are required to optimise the pre-clinical efficacy of this treatment and assess the translational potential of the combination strategy.

As aberrant metabolism drives T-ALL pathogenesis, pharmacological targeting of the dysregulated metabolic profile can elicit therapeutic benefit [2,5]. However, there are considerable difficulties in targeting cancer cell metabolism, which are attributed to severe toxicities and resistance. For example, the dependency of various cancers on complex I was exploited and led to a recently developed pharmacological inhibitor, IACS-010759 [8]. Despite demonstrating promising *in vitro* and preclinical results [5], clinical trials were stopped due to severe drug-associated toxicities and a limited therapeutic window [8]. Another example is the glutamine antagonist, 6-diazo-5-ox-l-norleucine (DON), which has been associated with gastrointestinal toxicity, thus impairing its clinical development [34]. Therefore, the rationale for drug repurposing - particularly T2D agents that target metabolism - is not only cost-driven, but is the established safety profile resulting in the circumvention or limitation of associated toxicities.

Unlike pharmacological targeting, attention has recently turned towards adopting dietary manipulation in cancer therapy, particularly serine restriction [[35], [36], [37]]. This opens up the intriguing possibility of coupling metabolic modulation, such as cana therapy, and dietary restriction in T-ALL. A further advantage could be extended to those patients who relapse, whereby resistance to therapy is associated with metabolic rewiring in T-ALL [38]. Inclusion of metabolic modulators as adjuvants overcomes resistance to agents such as glucocorticoids [39] and anti-NOTCH1 therapy [38]. Here, our data highlight the plausibility of coupling a metabolic modulator (cana) in addition to dietary restriction (serine/glycine) to enhance anti-leukaemic effects.

In conclusion, our manuscript demonstrates that the clinically approved T2D drug cana has anti-leukaemic properties and provides a foundation for the clinical development of cana for the treatment of T-ALL.

## 4 Materials and methods

### 4.1 Cell line maintenance and culture

T-ALL cell lines Jurkat, CCRF-CEM, DND-41, HPB-ALL, CUTLL1 and Loucy were obtained from the laboratory of Marc Mansour (UCL). RPMI8402 and TALL-1 were a kind gift from Cellestia. All cell lines were maintained in RPMI1640 supplemented with 10 % fetal bovine serum, 2 mM l-glutamine and 100U pen/100 μg/mL strep. For serine and glycine restriction, cells were cultured in a custom formulation of RPMI lacking serine and glycine obtained from The Francis Crick Institute and supplemented with 10 % dialysed FBS (Gibco), 400 μM l-serine, 400 μM glycine and 2 mM l-glutamine.

Cell lines routinely tested negative for mycoplasma infection. Cells were cultured at a density of 0.1 × 10^6^ cells/mL (PTEN^−^; Jurkat, RPMI8402, CCRF-CEM), 0.2 × 10^6^ cells/mL (PTEN^+^; DND-41, HPB-ALL, CUTLL1) and 0.4 × 10^6^ cells/mL (NOTCH1-independent; TALL-1, Loucy) for 72 h in the presence or absence of cana (0–40 μM; Cambridge Bioscience). Dapagliflozin and empagliflozin were obtained from Combi-Blocks and Cambridge Bioscience respectively, and used at 30 μM. The PHGDH inhibitor, BI-4916, was obtained from MedChemExpress and used at a final concentration of 15 μM. Cell counts were performed using a Countess automated cell counter (Invitrogen).

### 4.2 Primary cell isolation, maintenance and culture

Human naïve CD4+ T-cells were isolated as follows. PBMCs were isolated by density gradient centrifugation using Lymphoprep™ (StemCell Technologies). CD4+ naïve T-cells were then purified by negative selection using automated magnetic cell separation (Miltenyi; 130-094-131). Cells were activated in the presence of plate-bound anti-CD3 (2 μg/ml; OKT3; Biolegend) and soluble anti-CD28 (20 μg/ml; 28.2; Biolegend) or left unstimulated. Cultures were supplemented with 10 % FBS following 3 h incubation, to avoid impaired T-cell activation. Naïve CD4+ T-cells were cultured in the presence and absence of cana (30 μM) for 24 h, and downstream viability assessed.

Deidentified primary T-ALL samples were obtained from the University of Alabama at Birmingham (IRB-300009609, IRB-160422003) with informed consent per institutional guidelines and the Declaration of Helsinki. The cells were cultured in a custom formulation of RPMI lacking serine and glycine as described above.

### 4.3 Flow cytometry

Flow cytometry analysis was performed on T-ALL cell lines with DMSO vehicle control or 30 μM cana for 72 h. For mitochondrial ROS we utilised MitoSOX Red (5 μM; ThermoFisher) for 20 min at 37 °C. Dead cell monitoring and exclusion was performed using DRAQ7 (1 μM; BioStatus). Cells were acquired (Novocyte, ACEA) and downstream analysis was performed with FlowJo version 10, (TreeStar, USA). T-ALL proliferation was monitored using carboxyfluorescein succinimidyl ester (CFSE; Invitrogen).

For cell cycle analysis, cells were treated with DMSO vehicle control or 30 μM cana for 72 h. Cells were centrifuged at 500 × *g* for 5 min. Cells were washed in PBS and centrifuged at 500 × *g* for 5 min. Cells were fixed in 70 % ethanol on ice by resuspending the cells in 1 mL ice-cold ethanol, adding drop wise and gently mixing using a vortex at 1800 rpm. Cells were fixed at 4 °C for at least 2 h. Following fixation, the cells were brought to room temperature and gently resuspended. Cells were centrifuged at 850 × *g* for 5 min, before washing in PBS. Following washing, cells were resuspended in 500 μL Fx Cycle™ PI/RNase staining solution (Invitrogen). Cells were incubated for 20–30 min at 37 °C in the dark before analysis on the flow cytometer.

### 4.4 Extracellular flux analysis

Metabolic analysis of T-ALL cell lines was carried out using the Seahorse Extracellular Flux Analyzer (Agilent Technologies). Cells were resuspended in Seahorse XF assay media supplemented with 10 mM glucose, 1 mM sodium pyruvate and 2 mM l-glutamine (all Agilent Technologies). Cells were seeded at 0.2 × 10^6^/well onto a Cell-Tak (Corning) coated microplate allowing for immediate adhesion. To identify the immediate metabolic perturbations of gliflozins, T-ALL cell lines were injected initially with either cana, dapagliflozin or empagliflozin (all 30 μM). Corresponding OCR/ECAR changes were monitored for the duration of the experiment. For the long-term metabolic perturbations, Jurkat, CCRF-CEM, DND-41 or HPB-ALL cells were cultured with cana for 72 h. Cells were resuspended and seeded as above and a downstream mitochondrial stress or glycolytic rate assay were performed with injections of oligomycin (1 μM), FCCP (1 μM), antimycin A (1 μM), rotenone (1 μM) and monensin (20 μM) or antimycin A (1 μM), rotenone (1 μM) and 2-deoxy-d-glucose (50 mM; all injections obtained from Merck), respectively.

### 4.5 Stable isotope tracer analysis (SITA) by GC-MS

#### 4.5.1 GC-MS analysis of ^13^C metabolites

Gas chromatography coupled to mass spectrometry (GC-MS) was performed on T-ALL cells using previously described methods [[40], [41], [42]]. T-ALL cell lines (Jurkat and DND-41) were initially cultured for 16 h in the presence or absence of cana (30 μM). Cells were then washed with PBS and re-cultured in either glucose or glutamine free RPMI containing 10 % dialysed FBS and uniformly labeled ^13^C_6_-glucose or ^13^C_5_-glutamine, respectively (Cambridge Isotope Laboratories). Cells (4 × 10^6^/well in 6 well plates) were cultured in ^13^C-containing medium for up to 8 h with or without cana (30 μM). Metabolites were extracted using ice-cold 80 % methanol, sonicated, and then d-myristic acid was added (750 ng/sample) as an internal standard. Dried samples were dissolved in 30 μL methozyamine hydrochloride (10 mg/ml) in pyridine and derivatized as tert-butyldimethylsily (TBDMS) esters using 70 μL N-(*tert*-butyldimethylsilyl)-N-methyltrifluoroacetamide (MTBSTFA) [43].

For metabolite analysis, an Agilent 5975C GC/MS equipped with a DB-5MS + DG (30 m × 250 μm x 0.25 μm) capillary column (Agilent J&W, Santa Clara, CA, USA) was used. All data were collected by electron impact set at 70 eV. A total of 1 μL of the derivatized sample was injected in the GC in splitless mode with inlet temperature set to 280 °C, using helium as a carrier gas with a flow rate of 1.5512 mL/min (rate at which myristic acid elutes at 17.94 min). The quadrupole was set at 150 °C and the GC/MS interface at 285 °C. The oven program for all metabolite analyses started at 60 °C held for 1 min, then increased at a rate of 10 °C/min until 320 °C. Bake-out was at 320 °C for 10 min. Sample data were acquired both in scan (1–600 *m*/*z*) and selected ion monitoring (SIM) modes. Mass isotopomer distribution for cellular metabolites was determined using a custom algorithm developed at McGill University [44]. Briefly, the atomic composition of the TBDMS-derivatized metabolite fragments (M−57) was determined, and matrices correcting for natural contribution of isotopomer enrichment were generated for each metabolite. After correction for natural abundance, a comparison was made between non-labeled metabolite abundances (^12^C) and metabolite abundances which were synthesized from the ^13^C tracer. Metabolite abundance was expressed relative to the internal standard (d-myristic acid) and normalized to cell number.

### 4.6 LC-MS analysis of ^13^C metabolites

Metabolites were analyzed for relative abundance by high resolution accurate mass detection (HRAM) on two QExactive™ Orbitrap mass spectrometers (Thermo Fisher Scientific) coupled to Thermo Vanquish liquid chromatography systems as previously described [45]. Separate instruments were used for negative and positive mode analysis. For negative mode analysis, an Acquity T3 HSS (1.8 μm, 2.1 mm × 150 mm) column (Waters, Eschborn, Germany) was used for chromatographic separation and the elution gradient was carried out with a binary solvent system. Solvent A consisted of 3 % methanol, 10 mM tributylamine, and 15 mM acetic acid in water (pH 5.0 +/− 0.05) and solvent B was 100 % methanol. A constant flow rate of 200 μL min^−1^ was maintained and the linear gradient employed was as follows: 0–2.5 min 100 % A, 2.5–5 min increase from 0 to 20 % B, 5–7.5 min maintain 80 % A and 20 % B, 7.5–13 min increase from 20 to 55 % B, 13–15.5 min increase from 55 to 95 % B, 15.5–18.5 min maintain 5 % A and 95 % B, 18.5–19 min decrease from 95 to 0 % B, followed by 6 min of re-equilibration at 100 % A. The heater temperature was set to 400 °C and ion spray voltage was set to 2.75 kV. The column temperature was maintained at 25 °C and sample volumes of 10 μL were injected. A 22-minute full-scan method was used to acquire data with *m/z* scan range from 80 to 1200 and resolution of 70,000. The automatic gain control (AGC) target was set at 1e6 and the maximum injection time was 500 ms. For positive mode analysis, an Atlantis T3 (3 μm, 2.1 mm ID × 150 mm) column (Waters) was used and the elution gradient was carried out with a binary solvent system Solvent A consisted of 0.1 % acetic acid and 0.025 % heptafluorobutyric acid in water and solvent B was 100% acetonitrile. A constant flow rate of 400 μL min−1 was maintained and the linear gradient employed was as follows: 0–4 min increase from 0 to 30 % B, 4–6 min from 30 to 35 % B, 6–6.1 min from 35 to 100 % B and hold at 100 % B for 5 min, followed by 5 min of re-equilibration. The heater temperature was set to 300 °C and the ion spray voltage was set to 3.5 kV. The column temperature was maintained at 25 °C and sample volumes of 10 μL were injected. An 11-minute full-scan method was used to acquire data with *m/z* scan range from 70 to 700 and resolution of 70,000. The automatic gain control (AGC) target was set at 1e6 and the maximum injection time was 250 ms. Instrument control and acquisition was carried out by Xcalibur 2.2 software (Thermo Fisher Scientific).

For ^15^N-glutamine and ^13^C_3_^15^N-serine tracing, DND-41 and Jurkat cells were initially cultured for 16 h in the presence or absence of cana (30 μM). Cells were then washed with PBS and re-cultured in either serine and glycine or glutamine free RPMI containing 10% dialysed FBS and uniformly labeled ^13^C_3_^15^N-serine (608130; Merck) or ^15^N-glutamine (NLM-557; Cambridge Isotope Laboratories) for 8 h prior to extraction. All experimental conditions were cultured with 10% dialysed FBS. Duplicate plates were used for cell counting to normalize the LC-MS analysis by cell number. Cells were extracted with 1 mL of ice-cold extraction buffer (methanol/acetonitrile/water at a 50/30/20 ratio) per 2 × 10^6^ cells, vortexed and spun down at 15000 × *g* for 12 min at 4 °C. Supernatants were used for LC-MS analysis. Analytes were separated using hydrophilic interaction liquid chromatography with a SeQuant ZIC-pHILIC column (2.1 3 150 mm, 5 mm; Merck) and detected with high-resolution, accurate-mass mass spectrometry using an Q Exactive orbitrap from Thermofisher in line with a Vanquish autosampler and a Vanquish pump (Thermofisher). The elution buffers were acetonitrile for buffer A and 20 mM (NH_4_)_2_CO_3_ and 0.1 % NH_4_OH in H2O for buffer B. A linear gradient was programmed starting from 80 % buffer A and ending at 20 % buffer A after 20 min, followed by wash (20 % buffer A) and re-equilibration (80 % buffer A) steps with a flow rate of 300 μl min^−1^. The mass spectrometer was fitted with an electrospray-ionization probe and operated in full-scan and polar-switching mode with the positive voltage at 3.5 kV and negative voltage at 2.5 kV. Normalization was obtained with total ion count and with the presence of internal standard, D_10_ Leucine (DLM-567) from Cambridge Isotope Laboratories.

### 4.7 Glutamate dehydrogenase activity assay

Jurkat cells (1 × 10^6^/mL) were cultured in the presence or absence of cana (30 μM) for 24 h. Cells were then harvested, resuspended in 1 mL cold PBS, counted and normalised in GDH buffer accordingly. GDH activity was then assessed using a glutamate dehydrogenase activity assay kit (Abcam; ab102527).

### 4.8 Lactate assay

Extracellular l-lactate concentrations were measured using l-Lactate Assay Kit I (Eton Bioscience, USA) according to the manufacturer's instructions. Supernatants were diluted to an appropriate concentration prior to addition of l-Lactate Assay Solution and incubation at 37 °C for 30 min. Absorbance was measured at 490 nm after the addition of acetic acid (0.5 M; Merck) to each well and corrected to the blank.

### 4.9 Immunoblot

Lysate proteins were quantified, denatured and separated using SDS-polyacrylamide gel electrophoresis. Polyvinylidene difluoride membranes were probed with antibodies targeting phospho-acetyl-CoA carboxylase (pACC; Ser79; 3661), total ACC (3676), phospho-AMPK (pAMPK; Thr172; 2535), total AMPK (2532), phospho-S6 ribosomal protein (pS6; Ser235-236; 4858) and total S6 (2217). All antibodies were purchased from Cell Signaling and used at a 1:1000 dilution. Protein loading was evaluated and normalized using β-actin (ab8226; Abcam). Densitometry on non-saturated immunoblots was measured using ImageJ software (FIJI).

### 4.10 Proteomic analysis

#### 4.10.1 TMT Labelling, High pH reversed-phase chromatography and Phospho-peptide enrichment

Aliquots of 100 μg of each sample were digested with trypsin (2.5 μg trypsin per 100 μg protein; 37 °C, overnight), labelled with Tandem Mass Tag (TMTpro) sixteen plex reagents according to the manufacturer's protocol (Thermo Fisher Scientific) and the labelled samples pooled.

For the total proteome analysis, an aliquot of 50 μg of the pooled sample was desalted using a SepPak cartridge according to the manufacturer's instructions (Waters, Milford). Eluate from the SepPak cartridge was evaporated to dryness and resuspended in buffer A (20 mM ammonium hydroxide, pH 10) prior to fractionation by high pH reversed-phase chromatography using an Ultimate 3000 liquid chromatography system (Thermo Fisher Scientific). In brief, the sample was loaded onto an XBridge BEH C18 Column (130 Å, 3.5 μm, 2.1 mm × 150 mm; Waters, UK) in buffer A and peptides eluted with an increasing gradient of buffer B (20 mM Ammonium Hydroxide in acetonitrile, pH 10) from 0 to 95 % over 60 min. The resulting fractions (20 in total) were evaporated to dryness and resuspended in 1 % formic acid prior to analysis by nano-LC MSMS using an Orbitrap Fusion Lumos mass spectrometer (Thermo Scientific).

#### 4.10.2 Nano-LC mass spectrometry

High pH RP fractions (Total proteome analysis) or the phospho-enriched fractions (Phospho-proteome analysis) were further fractionated using an Ultimate 3000 nano-LC system in line with an Orbitrap Fusion Lumos mass spectrometer (Thermo Scientific). In brief, peptides in 1 % (vol/vol) formic acid were injected onto an Acclaim PepMap C18 nano-trap column (Thermo Scientific). After washing with 0.5 % (vol/vol) acetonitrile 0.1 % (vol/vol) formic acid peptides were resolved on a 250 mm × 75 μm Acclaim PepMap C18 reverse phase analytical column (Thermo Scientific) over a 150 min organic gradient, using 7 gradient segments (1–6 % solvent B over 1min., 6–15 % B over 58min., 15–32 %B over 58min., 32–40 %B over 5min., 40–90 %B over 1min., held at 90 %B for 6min and then reduced to 1 %B over 1min.) with a flow rate of 300 nl min^−1^. Solvent A was 0.1 % formic acid and Solvent B was aqueous 80 % acetonitrile in 0.1 % formic acid. Peptides were ionized by nano-electrospray ionization at 2.0 kV using a stainless-steel emitter with an internal diameter of 30 μm (Thermo Scientific) and a capillary temperature of 300 °C.

All spectra were acquired using an Orbitrap Fusion Lumos mass spectrometer controlled by Xcalibur 3.0 software (Thermo Scientific) and operated in data-dependent acquisition mode using an SPS-MS3 workflow. FTMS1 spectra were collected at a resolution of 120,000, with an automatic gain control (AGC) target of 200,000 and a max injection time of 50 ms. Precursors were filtered with an intensity threshold of 5000, according to charge state (to include charge states 2–7) and with monoisotopic peak determination set to Peptide. Previously interrogated precursors were excluded using a dynamic window (60s +/−10ppm). The MS2 precursors were isolated with a quadrupole isolation window of 0.7m/z. ITMS2 spectra were collected with an AGC target of 10,000, max injection time of 70 ms and CID collision energy of 35 %.

For FTMS3 analysis, the Orbitrap was operated at 50,000 resolution with an AGC target of 50,000 and a max injection time of 105 ms. Precursors were fragmented by high energy collision dissociation (HCD) at a normalised collision energy of 60 % to ensure maximal TMT reporter ion yield. Synchronous Precursor Selection (SPS) was enabled to include up to 10 MS2 fragment ions in the FTMS3 scan.

The raw data files were processed and quantified using Proteome Discoverer software v2.4 (Thermo Scientific) and searched against the UniProt Human database (downloaded January 2022: 178486 entries) using the SEQUEST HT algorithm. Peptide precursor mass tolerance was set at 10ppm, and MS/MS tolerance was set at 0.6Da. Search criteria included oxidation of methionine (+15.995Da), acetylation of the protein N-terminus (+42.011Da) and Methionine loss plus acetylation of the protein N-terminus (−89.03Da) as variable modifications and carbamidomethylation of cysteine (+57.0214) and the addition of the TMTpro mass tag (+304.207) to peptide N-termini and lysine as fixed modifications. For the Phospho-proteome analysis, phosphorylation of serine, threonine and tyrosine (+79.966) was also included as a variable modification. Searches were performed with full tryptic digestion and a maximum of 2 missed cleavages were allowed. The reverse database search option was enabled and all data was filtered to satisfy false discovery rate (FDR) of 5 %.

### 4.11 Lentiviral transduction of DND-41 cells

1 × 10^6^ DND-41 cells were centrifuged with pHAGE PGK-GFP-IRES-LUC-W (Addgene) Lentivirus and 10 μg/mL polybrene (Sigma-Aldrich) at 200 g for 30 min, then incubated for 24 h after which media was replaced. Following 3 passages, GFP + cells were sorted at 352 V and gated for a GFP signal exceeding 10^3^ using a BD FACSAria Fusion Cell Sorter. They were further expanded and tested for luciferase signal strength using Steady-Glo (Promega), and finally for mycoplasma before transplantation into mice.

### 4.12 Xenografts

5 × 10^6^ DND-41 GFP-Luc cells were transplanted via tail vein injection into 15 male NOD.Cg-Prkdc^SCID^Il2rg^tm1Wjl^/SzJ (NSG) mice. Mice were bred in house and original breeding pairs obtained from Charles River. Sterile IVC cages were used for housing. A 7 day engraftment period was allowed before mice were randomly placed on + SG (n = 7) (T-5BQS-1816652-203 Baker AA Diet) or -SG diet (n = 8) (T-5BQT-1816653-203 Baker AA Diet) (Test Diet, International Product Supplies). Mice were maintained on this diet for 18 days. The caloric value and total amino acid content was equal between the diets. Mice were allowed to adjust to the diet for 7 days before starting treatment with 30 mg/kg cana (n = 7) or the vehicle 0.5 % (Hydroxypropyl)methyl Cellulose (n = 8) (H7509-25G, Sigma-Aldrich) via gavage for two blocks of 5 days with 2 days of break in between.

As this diet can cause moderate weight loss, mice were monitored daily for weight changes and supplemented with 20 % sucrose (Thermo-Fisher) in their drinking water from 9 days post-transplant until the end of diet and drug treatment.

Leukaemic burden was monitored by IVIS imaging immediately after, 3 days post-transplant, and then every 7 days. IVIS Spectrum *in vivo* optical imaging system (PerkinElmer, Waltham, Massachusetts) was used with default acquisition settings (open emission filter, blocked excitation filter, medium binning, f stop 1), a field of view (FOV) of 22.8 cm, acquisition times of 4s for dorsal imaging and 9s for ventral imaging. Beetle Luciferin (potassium salt; Promega; #E1602) was made up at 15 mg/mL sterile DPBS, pH 7.0 (Gibco #14190-094) and 100uL injected per mouse. 3 mice were excluded from analysis of impact of treatment on disease burden (2 due to non-engraftment on IVIS imaging and 1 culled early due to weight loss unrelated to leukaemic burden). All animals were sacrificed once they reached clinical endpoint. All animal work was approved by the Ethical Review Process (University of Glasgow) and undertaken in line with the UK Animals (Scientific Procedures) Act of 1986 (PPL 5432144) and the EU directive 2010.

### 4.13 Data analysis

Statistical analysis was performed using GraphPad Prism version 10 (USA). Data are represented as the mean ± or + standard error of the mean (SEM). All experiments have replicate sample sizes of at least n = 3 and significant values were taken as ∗ p ≤ 0.05, ∗∗p ≤ 0.01, ∗∗∗p ≤ 0.001 or ∗∗∗∗p ≤ 0.0001.

## CRediT authorship contribution statement

**Fernando M. Ponce-Garcia:** Writing – review & editing, Methodology, Investigation, Formal analysis, Data curation. **Yasmin R. Jenkins:** Writing – review & editing, Investigation, Formal analysis, Data curation. **Victoria D. Assmann:** Writing – review & editing, Investigation, Formal analysis, Data curation. **Silpita Paul:** Formal analysis, Data curation. **Nitesh D. Sharma:** Formal analysis, Data curation. **Catherine Moore:** Formal analysis, Data curation. **Eric H. Ma:** Writing – review & editing, Methodology, Investigation, Formal analysis, Data curation. **Paraskevi Diamanti:** Formal analysis, Data curation. **Marc Hennequart:** Writing – review & editing, Methodology, Investigation, Formal analysis, Data curation. **Julianna Blagih:** Writing – review & editing, Formal analysis, Data curation. **Le Le:** Formal analysis, Data curation. **Benjamin J. Jenkins:** Formal analysis, Data curation. **Sophie Rouvray:** Formal analysis, Data curation. **James G. Cronin:** Investigation, Formal analysis, Data curation. **Russell G. Jones:** Resources, Methodology, Investigation, Data curation. **Marc Mansour:** Supervision, Resources, Methodology, Conceptualization. **Allison Blair:** Supervision, Funding acquisition, Formal analysis, Data curation, Conceptualization. **Christina Halsey:** Writing – review & editing, Supervision, Resources, Methodology, Investigation, Data curation, Conceptualization. **Ksenia Matlawska-Wasowska:** Writing – review & editing, Supervision, Resources, Investigation, Formal analysis, Data curation. **Daniel Herranz:** Writing – review & editing, Supervision, Resources, Methodology, Investigation, Formal analysis, Data curation, Conceptualization. **Emma E. Vincent:** Writing – review & editing, Writing – original draft, Supervision, Methodology, Investigation, Funding acquisition, Formal analysis, Data curation, Conceptualization. **Nicholas Jones:** Writing – review & editing, Writing – original draft, Visualization, Validation, Supervision, Methodology, Investigation, Funding acquisition, Formal analysis, Data curation, Conceptualization.

## Declaration of competing interest

The authors declare that they have no known competing financial interests or personal relationships that could have appeared to influence the work reported in this paper.

## Data Availability

The mass spectrometry proteomics data have been deposited to the ProteomeXchange Consortium via the PRIDE partner repository with the dataset identified PXD069587. Source data are available to download. Data will be made available on request.
